# Combined dimercaptosuccinic acid and zinc treatment in neurological Wilson’s disease patients with penicillamine-induced allergy or early neurological deterioration

**DOI:** 10.1042/BSR20200654

**Published:** 2020-08-18

**Authors:** Xiao-Qun Zhu, Liang-Yong Li, Wen-Ming Yang, Yu Wang

**Affiliations:** 1 Department of Neurology, The First Affiliated Hospital of Anhui Medical University, Hefei 230022, China; 2 Department of Neurology, The First Affiliated Hospital of Anhui University of Traditional Chinese Medicine, Hefei 230031, China

**Keywords:** Allergy, D-penicillamine, Dimercaptosuccinic acid, Neurological deterioration, Wilson's disease, Zinc

## Abstract

The clinical data of safety and efficacy of a combined treatment with dimercaptosuccinic acid (DMSA) and Zinc with 2 years’ follow-up in 60 neurological Wilson’s disease (WD) patients was retrospectively analyzed. All the patients included in the present study were newly diagnosed and initialized with D-penicillamine (DPA) treatment but were found to have either neurological deterioration or allergy, and their treatment was switched to a combined treatment of DMSA and Zinc. Fifty-one patients (85%) had the neurological symptoms improved 1 and 2 years after treatment, 7 (11.67%) experienced a stable neurological condition, and 2 (3.33%) suffered deterioration of neurological symptoms. No early neurological deterioration was observed in all patients. Twenty-five percent patients experienced mild adverse reactions which did not require a discontinuation of the DMSA and Zinc treatment. Our study confirmed the safety and efficacy of the combined DMSA and Zinc therapy as an initial and probably long-term treatment in neurological WD patients.

## Introduction

Wilson’s disease (WD) is an autosomal recessive disorder of copper metabolism resulting in the pathological accumulation of copper in various tissues and organs, predominantly in the liver, brain, cornea, and kidneys. Toxic copper accumulation in these tissues and organs may contribute to a variety of clinical conditions with predominant neurological, psychiatric, and hepatic symptoms [1]. WD is lethal if left untreated. Current treatment regimens include copper chelators promoting copper excretion such as the commonly used D-penicillamine (DPA), trientine,and Zinc salts [1]. Zinc salt reducing copper absorption is usually used in combination with DPA due to its incapability to excrete abundant copper and this combination therapy was reported to have significantly higher mortality rate [2]. Although various studies have documented its efficacy, DPA has serious toxicity, especially causing neurological deterioration in ∼10–50% of WD patients during the initial phase of treatment [3–5], and DPA has been commonly reported to be able to cause serious allergy including epidermolysis bullosa acquisita [6–8]. As an alternative, trientine or tetrathiomolybdate has been introduced, but their high medical cost and non-availability restrict their applicability in China [9]. Thus, other safe and effective copper chelator is of significant importance for those WD patients who had early neurological deterioration after penicillamine treatment and for those who were allergic to penicillamine.

Dimercaptosuccinic acid (succimer; DMSA), a water-soluble analog of dimercaprol, has been used as an antidote for heavy metal toxicity for decades since 1950s [10]. DMSA can form complexes with copper ions and oral DMSA significantly increased the urinary copper excretion [11,12]. DMSA was first used as a copper chelator for WD in China [13,14] and there are substantial experiences with the use of DMSA for WD treatment in China [9,15,16]. However, previous studies had never focused on neurological WD and lacked the observations of liver function and counts of leukocytes and platelet and the long-term follow-up. Zinc salt and a chelating agent (chelator) are usually utilized in combination for metallothionein induction to block copper uptake and elimination of excess copper, respectively [17]. And this combined therapy has been demonstrated to be effective and safe for neurological WD patients, and superior to Zinc monotherapy [2,18,19]. Thus, DSMA is usually used in combination with Zinc salt for WD patients in our center.

In the present study, we retrospectively analyzed the efficacy of a combined DSMA and Zinc treatment in 60 neurological WD patients who had either a history of early neurological deterioration after DPA treatment or a history of allergy to DPA, and its safety and efficacy with 2 years’ follow-up were outlined.

## Methods

### Inclusion and exclusion criteria

Medical records of WD patients with either DPA-induced neurological deterioration or allergy to treatment in The First Affiliated Hospital of Anhui University of Traditional Chinese Medicine from 2013 to 2015 were retrospectively analyzed. The Institutional Review Board in the First Affiliated Hospital of Anhui Medical University and Anhui Medical University of Traditional Chinese Medicine approved the present study and waived the need for informed consent for clinical data of the patients to be used for retrospective analysis (ethics approval number: Quick-PJ 2017-09-01).

The diagnosis of WD was based on our previous standard [20], including characteristic clinical manifestations, positive family history, low serum ceruloplasmin (<0.2 g/l), elevated 24-h urinary copper (24-h UC) excretion (>100 µg/24 h), elevated liver copper (>250 µg/g dry weight), presence of a Kayser–Fleischer (K–F) ring, elevated 24-h UC excretion following the administration of 2× 500-mg doses of DPA (>1600 µg/24 h) and magnetic resonance imaging (MRI) of brain [21,22], and met the WD diagnostic criteria formed at the 8th International Meeting on Wilson’s disease [23,24].

For classification of WD types, symptoms scoring system was adopted and mildly modified from Litwin et al.’s papers [25,26]. Briefly, assessment of neurological symptoms ranged from 0 to 3 representing neurological impairment from completely normal (0) to severely impaired (3). Assessment of hepatic symptoms ranged from 0 to 3 representing completely normal (0), increased serum level of liver enzymes with no liver cirrhosis (1), compensated liver cirrhosis (2), decompensated liver cirrhosis or acute liver failure (3). Patients with neurological score ≥ 1 and hepatic score ≤1 were regarded as neurological WD, and patients with neurological score ≥1 and hepatic score ≥2 regarded as neurological and hepatic WD.

Patients included in the present study were those who were newly diagnosed with neurological WD and initialized with DPA treatment but were found to have either neurological deterioration or allergy, and their treatment was switched to combined DMSA and Zinc treatment. Patients with insufficient data, hepatic score ≥ 2, severe psychiatric disorders, pregnancy, lactating, and allergy to the trial medicine were excluded from the present study. Patients with long-term DPA treatment and later neurological deterioration were also excluded from the present study though their treatments were switched to combination therapy of DMSA and Zinc. Patients with severe allergy such as hypersensitivity pneumonitis and high fever were excluded to eliminate the possible influence on neurological scores.

### Data collection and analysis

Baseline characteristics including age, gender, K–F rings, ceruloplasmin (CER), white blood cell (WBC), and platelet (PLT) counts were recorded. Liver function represented with aspartate aminotransferase (AST), alanine aminotransferase (ALT), albumin (ALB), prothrombin time (PT), and international normalized ratio (INR) in serum or plasma and 24-h urine copper were recorded. The recorded Global Assessment Scale (GAS) [27] was used to access the changes of neurological symptoms for each patient. The GAS has now been well utilized in clinical assessment of WD demonstrating it to be an appropriate tool for monitoring disease progression and treatment response in WD patients. This scale contains two-tier scoring: WD-related disability and WD-related neurological dysfunction. A total score of 0 indicates absence of neurological sign and a total score ranging from 1 to 56 indicates the different severities of neurological impairment. Meanwhile, the frequency and type of complications were recorded. Here, the data were not available at consecutive time points for all the patients during the treatment but available before treatment and by 1 and 2 years after treatment. Thus, the data for comparison were recorded at the time before treatment and by 1 and 2 years after treatment.

### Statistical analysis

Statistical analyses were performed using a commercial statistical software package (SPSS for Windows, version 22.0). Data were expressed as mean ± SD. Paired *t* tests were used for statistical analyses. Differences were considered significant at *P*<0.05.

## Results

### Baseline characteristics of patients

Consequently, a total of 60 neurological WD patients were analyzed. Thirty-nine out of 60 patients were initially diagnosed and treated with DPA in other hospitals but had DPA-induced early neurological deterioration, and the duration of chelation therapy ranged from 1 to 3 months. Eleven out of the 39 cases suffered rapid deterioration in 2 weeks after DPA treatment. DPA was started at a low dose (0.125 g twice a day for adults, 0.125 g once a day for children) and gradually increased to routine dosage (0.25 g thrice a day) in 31 cases. Unfortunately, DPA was started at a routine dosage (0.25 g twice to thrice a day) in eight cases. After DPA-induced early neurological deterioration, DPA was stopped or the dose was reduced, but the neurological deterioration did not improve. The patients were then transferred to our center with confirmation of WD diagnosis with our standard and the treatment was switched to combination therapy of DMSA and Zinc. Twenty-one patients were allergic to DPA presenting with cutaneous rash or pemphigus-like cutaneous changes within days to 2 weeks of DPA treatment. The demographic and clinical features of the 60 patients are listed in Table 1. Thirty-four were males and 26 females. The mean age at onset and age at diagnosis were 20.12 ± 7.95 years (range 8–39) and 20.43 ± 7.99 years (range: 8–41), respectively. The neurological manifestations of the 60 WD patients included dysarthria, tremor, drooling, dystonia, incoordination, dysphagia, and spasticity. All patients had K–F rings and displayed low serum CER (<0.2 g/l) and elevated 24-h UC excretion (>100 µg/24 h). Brain MRI was found abnormal in all 60 patients primarily involving the basal ganglia, thalamus, brainstem, frontal lobe, and cerebellum.

**Table 1:** Demographic and clinical features of the WD patients

Patients (*n*)	60
Age at onset (years)	20.12 ± 7.95
Age at diagnosis (years)	20.43 ± 7.99
Gender (M/F)	34/26
Serum CER (g/l)	0.09 ± 0.01
24-h UC excretion (µg/24 h)	450.63 ± 148.98
K–F rings (*n*)	60
Neurologic (*n*)	60
Brain MRI abnormalities of WD (*n*)	60

### Treatment

The neurological WD patients in the present study were treated with combination therapy of DMSA and Zinc. The daily DMSA (20–25 mg/kg) was administered before meals in two divided doses, and daily Zinc (240 mg, 80 mg Zinc contained in one tablet of 560 mg Zinc Gluconate) administered after meals in three divided doses. The pediatric dose of DMSA is 15 mg/kg until 12 years of age. Meanwhile, patients were requested to take a low copper diet.

### Neurological outcomes

Fifty-one patients (85%) had the neurological symptoms improved 1 and 2 years after treatment, 7 (11.67%) cases experienced a stable neurological condition, and 2 (3.33%) suffered from deterioration of neurological symptoms. Deterioration of neurological symptoms 2 years after treatment was reported in two patients who did not stick to strict low copper diet during the new treatment. In addition, the value of GAS 2 years after treatment was mildly but not statistically decreased compared with ar after treatment (Table 2). No early neurological deterioration was observed within weeks to months of DMSA treatment in all 60 patients.

**Table 2:** Neurological symptom scoring before and after combined DMSA and Zinc treatment (mean ± SD)

Time	GAS
Before DMSA	16.55 ± 3.19
1 year after DMSA	11.67 ± 3.27^*^
2 years after DMSA	10.87 ± 3.42^*^

* *P*<0.01, compared with that before DMSA treatment.

### Blood cell counts

As shown in Table 3, the WBC and PLT counts stabilized with mild decrease 1 and 2 years after DMSA treatment. However, no statistically significant difference was found in WBC and PLT counts before and after treatments and no statistically significant difference 1 and 2 years after treatment (*P*>0.05).

**Table 3:** WBC and PLT counts before and after combined DMSA and Zinc treatment (mean ± SD)

Variables	Before DMSA	1 year after DMSA	2 years after DMSA
WBC (×10^9^/l)	6.56 ± 1.80	6.45 ± 1.50	6.19 ± 1.48
PLT (×10^9^/l)	207 ± 52.0	199.98 ± 52.1	192.60 ± 49.54

### Liver functions

The serum concentrations of ALT, AST, ALB, INR, and PT at start of therapy and by 1 and 2 years after treatment are presented in Table 4. The five markers implicating liver function (ALT, AST, ALB, INR, and PT) mildly decreased at 1 and 2 years after treatment. However, no statistically significant differences were found before and after treatment and no statistically significant difference 1 and 2 years after treatment (*P*>0.05). As for liver function, 54 WD patients (90%) remained stable, and 6 (10%) presented mild hepatic deterioration with mildly increased ALT and AST levels in four cases (6.67%) and mildly increased PT and INR levels in three cases (5%). No patient exhibited severely decompensated cirrhosis after treatment.

**Table 4:** Liver functions before and after combined DMSA and Zinc treatment (mean ± SD)

Variables	Before DMSA	1 year after DMSA	2 years after DMSA
ALT (U/l)	29.28 ± 7.94	28.58 ± 9.31	26.15 ± 9.19
AST (U/l)	26.25 ± 6.37	24.58 ± 7.54	22.62 ± 7.49
ALB	45.66 ± 2.62	46.53 ± 2.66	47.38 ± 2.99
PT (s)	11.67 ± 1.23	11.80 ± 1.85	11.56 ± 1.84
INR	1.08 ± 0.06	1.09 ± 0.07	1.10 ± 0.07

### 24-h UC

24-h UC excretion was sharply elevated after treatment (*P*<0.05) (Table 5). The value of 24-h UC by 2 years after treatment was mildly decreased, but still significantly higher than that by 1 year after treatment (*P*<0.05). No statistically significant difference in 24-h UC excretion was noted 1 and 2 years after treatment (*P*>0.05).

**Table 5:** 24-h UC excretion before and after combined DMSA and zinc treatment (mean ± SD)

Variables	Before DMSA	1 year after DMSA	2 years after DMSA
24-h UC excretion (µg/24 h)	450.63 ± 148.98	1617.01 ± 598.85^*^	1558.26 ± 613.72^*^

* *P*<0.01, compared with that before DMSA treatment.

### Side effects

Fifteen patients (25%) experienced adverse reactions in our study during the 2 years of follow-up. The types of side effects included neurological deterioration in two cases (3.33%), mild hepatic worsening in six cases (10%), poor appetite in three cases (5%), abdominal discomfort in two cases (3.33%), and joint pain in three cases (5%). However, all patients had better tolerance and no patient exhibited severely decompensated cirrhosis after treatment.

## Discussion

Our results showed that 85% of the neurological WD patients treated with DMSA and Zinc experienced an improved neurological condition paralleled by a significantly improved GAS score after 1 and 2 years follow-up. This improvement rate is higher than that of neurological WD patients treated with DPA monotherapy (67.9–69%) [2,28] and higher than that of neurological WD patients treated with DPA and Zinc combination therapy (78.4%) [29]. Here, the improvement rates were from literature reports of different follow-up durations. One literature report demonstrated a high improvement rate of 92% in neurological WD patients after 1 year treatment with DPA monotherapy, but the patients were all children with average age (9.06 ± 2.65) much younger than tients (20.43 ± 7.99) [30]. Another encouraging finding is that no early neurological deterioration was observed after the combined DMSA and Zinc treatment was started and the deterioration rate within the follow-up period was only 3.33% which is much lower than that (11.3–21.9%) induced by DPA [28,31] and much lower than that (26.3%) induced by Zinc combined with many other chelators [2]. Finally, the improvement rate induced by combined DMSA and Zinc treatment was much higher than that (11.8%) induced by Zinc monotherapy in Chinese patients with neurological WD [19]. This indicated that the combined DMSA and Zinc therapy may be used as an alternative treatment with high neurological improvement rate in neurological WD patients after DPA treatment failure. Other alternative agent, such as combination treatment of sodium dimercaptopropanesulfonate (DMPS) and Zinc, had also been tried in neurological WD patients after DPA treatment failure [19]. But its improvement rate (76.2%) was much lower and neurological deterioration rate after 1 year treatment much higher (19.0%) than those in our patients treated with combination of DMSA and Zinc (improvement rate 85%, and deterioration rate 3.33%), though the initial GAS score before treatment was lower than that of our patients (13.4 ± 5.6 vs. 16.55 ± 3.19) indicating the early neurological symptom was more severe in our patients [19]. The perfect therapeutic effect of DMSA could be due to its dramatic capacity of copper excretion through kidney as 24-h UC excretion was sharply elevated 1 and 2 years after DMSA treatment.

With respect to the adverse effects caused by the combined DMSA and Zinc treatment, only 25% of the WD patients experienced adverse reactions including neurological deterioration in two cases (3.33%), mild hepatic worsening in six cases (10%), poor appetite in three cases (5%), abdominal discomfort in two cases (3.33%), and joint pain in three cases (5%). These adverse effects caused by the combined DMSA and Zinc treatment seem much milder than the common adverse effects caused by DPA and many other chelating agents. The common adverse effects of DPA and other chelating agents are bone marrow depression, anemia/leukopenia, immunological lesions, and even paradoxical symptom worsening [4,31,32]. As for liver function, mild elevation of hepatic transaminase activities (ALT, AST) was reported in 14% patients with lead poisoning after DMSA therapy [33]. Others reported liver function normalized and even improved during DMSA therapy [12,34,35]. In current study, 54 WD patients remained stable, and 6 cases presented mild hepatic deterioration with mild increased ALT and AST levels in 4 cases, and mild increased PT and INR levels in 3 cases, and no patient exhibited severely decompensated cirrhosis after treatment. This is consistent with literature reports [12]. In addition, the WBC and PLT counts stabilized with mild decrease by 1 and 2 years after treatment without neutropenia in our study, which was similar to that of previous study [12]. Although other adverse effects including poor appetite (5%), abdominal discomfort (3.33%), and joint pain (5%) were observed, they were well-tolerated. In fact, the WBC and PLT counts stabilized, and the liver function remained stable in 90% cases. In general, DMSA treatment caused no clinically severe adverse effects and no laboratory findings requiring discontinuation of treatment in our neurological WD patients, as observed in severe lead poisoning patients [36].

### Study limitations

The main limitation of the present study was that it was a retrospective and observational but not a randomized control study, thus there was no comparison in efficacy and safety between the combined DMSA and Zinc treatment and the Zinc monotherapy. However, we have chosen to publish these data to share our experience of combined therapy with DMSA and Zinc as it had much higher neurological improvement rate and lower side effect rate compared with those from literatures.

For some patients, DMSA and Zinc doses were dynamically adjusted according to tolerance and liver enzyme changes during the treatment. We attempted to collect intermediate data of neurological score, blood cell counts, liver function, and 24-h UC excretion, but the collected data were inadequate for statistical analysis as they were available at different time points after treatment except for their availability before treatment and 1 and 2 years after treatment.

24-h UC and non-CER serum copper are important parameters for evaluating the efficacy of WD treatment [37,38]. It is recommended to maintain a 24-h UC excretion among a certain level a day based on body weight to achieve good efficacy and reduce the possibility of copper deficiency [39]. But these data were not available in this retrospective study as body weight and non-CER copper levels were not regularly measured during follow-up visits. However, no patient was reported to have hematological problems and neurological signs probably due to copper deficiency after the combined DMSA and Zinc treatment in our study and the PT and INR were stable during follow-up visits.

## Conclusions

The combination therapy of DMSA and Zinc effectively improved the neurological symptoms with a powerful de-coppering capacity in neurological WD patients who had a history of DPA-induced allergy or early neurological deterioration. In particular, only 25% of the patients experienced mild adverse reactions which did not require discontinuation of the treatment. Our study confirmed the safety and efficacy of the combined DMSA and Zinc therapy as an initial and long-term treatment in neurological WD patients. A longer follow-up would be useful to further estimate the clinical value of the combined DMSA and Zinc therapy in WD patients.

## Abbreviations

ALB: albumin
ALT: alanine aminotransferase
AST: aspartate aminotransferase
CER: ceruloplasmin
DMSA: dimercaptosuccinic acid
DPA: D-penicillamine
GAS: Global Assessment Scale
INR: international normalized ratio
MRI: magnetic resonance imaging
K–F: Kayser–Fleischer
PLT: platelet
PT: prothrombin time
WBC: white blood cell
WD: Wilson’s disease
24-h UC: 24-h urinary copper

## References

[1] Harada M. (2014) Pathogenesis and management of Wilson disease. Hepatol. Res. 44, 395–402 10.1111/hepr.12301 24450973

[2] Chen J.C., Chuang C.H., Wang J.D. and Wang C.W. (2015) Combination therapy using chelating agent and zinc for Wilson’s disease. J. Med. Biol. Eng. 35, 697–708 10.1007/s40846-015-0087-7 26692828 PMC4666238

[3] Ranjan A., Kalita J., Kumar V. and Misra U.K. (2015) MRI and oxidative stress markers in neurological worsening of Wilson disease following penicillamine. Neurotoxicology 49, 45–49 10.1016/j.neuro.2015.05.004 26004675

[4] Kalita J., Kumar V., Chandra S., Kumar B. and Misra U.K. (2014) Worsening of Wilson disease following penicillamine therapy. Eur. Neurol. 71, 126–131 10.1159/000355276 24356057

[5] Veen C., van den Hamer C.J. and de Leeuw P.W. (1991) Zinc sulphate therapy for Wilson’s disease after acute deterioration during treatment with low-dose D-penicillamine. J. Intern. Med. 229, 549–552 10.1111/j.1365-2796.1991.tb00395.x 2045766

[6] Vajdi T., Lee W.W. and Paravar T. (2016) Penicillamine-associated cutis laxa and milia en plaque - case report and review of cutaneous changes associated with penicillamine. Dermatol. Online J. 22, 13030 27617526

[7] Ingen-Housz-Oro S., Grootenboer-Mignot S., Ortonne N., Nahon S., Horvath J., Bernardeschi C. et al. (2014) Epidermolysis bullosa acquisita-like eruption with anticollagen VII autoantibodies induced by D-penicillamine in Wilson disease. Br. J. Dermatol. 171, 1574–1576 10.1111/bjd.13153 24888462

[8] Ishak R. and Abbas O. (2013) Penicillamine revisited: historic overview and review of the clinical uses and cutaneous adverse effects. Am. J. Clin. Dermatol. 14, 223–233 10.1007/s40257-013-0022-z 23605177

[9] Li W.J., Chen C., You Z.F., Yang R.M. and Wang X.P. (2016) Current drug managements of Wilson’s disease: from west to east. Curr. Neuropharmacol. 14, 322–325 10.2174/1570159X14666151130222427 26639459 PMC4876588

[10] Froitzheim G. and Olligs H. (1954) Experimental studies on the treatment of mercurial poisoning with alpha,alpha 1-dimercaptosuccinic acid. Dermatol. Wochenschr. 129, 497–500 13173207

[11] Cao Y., Skaug M.A., Andersen O. and Aaseth J. (2015) Chelation therapy in intoxications with mercury, lead and copper. J. Trace Elem. Med. Biol. 31, 188–192 10.1016/j.jtemb.2014.04.010 24894443

[12] Bradberry S. and Vale A. (2009) Dimercaptosuccinic acid (succimer; DMSA) in inorganic lead poisoning. Clin. Toxicol. (Phila.) 47, 617–631 10.1080/15563650903174828 19663612

[13] Yang R. (1990) Clinical analysis of 418 patients with Wilson’s disease in traditional Chinese medicine and Western medicine therapy. Zhong Xi Yi Jie He Za Zhi 10, 134–136, 2165877

[14] Li W.J., Wang J.F. and Wang X.P. (2013) Wilson’s disease: update on integrated Chinese and Western medicine. Chinese J. Integr. Med. 19, 233–240 10.1007/s11655-012-1089-8 22610954

[15] Ren M. and Yang R. (1997) Clinical curative effects of dimercaptosuccinic acid on hepatolenticular degeneration and the impact of DMSA on biliary trace elements. Chin. Med. J. (Engl.) 110, 694–697 9642327

[16] Ren M.S., Zhang Z., Wu J.X., Li F., Xue B.C. and Yang R.M. (1998) Comparison of long lasting therapeutic effects between succimer and penicillamine on hepatolenticular degeneration. World J. Gastroenterol. 4, 530–532 10.3748/wjg.v4.i6.530 11819363 PMC4723446

[17] Schilsky M.L. (2001) Treatment of Wilson’s disease: what are the relative roles of penicillamine, trientine, and zinc supplementation? Curr. Gastroenterol. Rep. 3, 54–59 10.1007/s11894-001-0041-4 11177695

[18] Weiss K.H., Gotthardt D.N., Klemm D., Merle U., Ferenci-Foerster D., Schaefer M. et al. (2011) Zinc monotherapy is not as effective as chelating agents in treatment of Wilson disease. Gastroenterology 140, 1189.e1181–1198.e1181 10.1053/j.gastro.2010.12.034 21185835

[19] Chen D., Zhou X., Hou H., Feng L., Liu J., Liang Y. et al. (2016) Clinical efficacy of combined sodium dimercaptopropanesulfonate and zinc treatment in neurological Wilson’s disease with D-penicillamine treatment failure. Ther. Adv. Neurol. Disord. 9, 310–316 10.1177/1756285616641598 27366238 PMC4916523

[20] Li L.Y., Yang W.M., Chen H.Z., Wu Y.H., Fang X., Zhang J. et al. (2015) Successful splenectomy for hypersplenism in Wilson’s disease: a single center experience from China. PLoS ONE 10, e0124569 10.1371/journal.pone.0124569 25910248 PMC4409367

[21] Rosencrantz R. and Schilsky M. (2011) Wilson disease: pathogenesis and clinical considerations in diagnosis and treatment. Semin. Liver Dis. 31, 245–259 10.1055/s-0031-1286056 21901655

[22] Ferenci P. (2004) Review article: diagnosis and current therapy of Wilson’s disease. Aliment. Pharmacol. Ther. 19, 157–165 10.1046/j.1365-2036.2003.01813.x 14723607

[23] Ferenci P., Caca K., Loudianos G., Mieli-Vergani G., Tanner S., Sternlieb I. et al. (2003) Diagnosis and phenotypic classification of Wilson disease. Liver Int. 23, 139–142 10.1034/j.1600-0676.2003.00824.x 12955875

[24] Rathnayake G., Saad M., Choy K.W. and Doery J.C. (2016) Diagnosis of Wilson’s disease - a 20 year audit. Pathology 48, S90

[25] Litwin T., Gromadzka G. and Czlonkowska A. (2012) Gender differences in Wilson’s disease. J. Neurol. Sci. 312, 31–35 10.1016/j.jns.2011.08.028 21917273

[26] Litwin T., Gromadzka G., Czlonkowska A., Golebiowski M. and Poniatowska R. (2013) The effect of gender on brain MRI pathology in Wilson’s disease. Metab. Brain Dis. 28, 69–75 10.1007/s11011-013-9378-2 23315358 PMC3562549

[27] Aggarwal A. and Bhatt M. (2013) Update on Wilson disease. Int. Rev. Neurobiol. 110, 313–348 10.1016/B978-0-12-410502-7.00014-4 24209444

[28] Walshe J.M. and Yealland M. (1993) Chelation treatment of neurological Wilson’s disease. QJM 86, 197–204 8369040

[29] Bruha R., Marecek Z., Pospisilova L., Nevsimalova S., Vitek L., Martasek P. et al. (2011) Long-term follow-up of Wilson disease: natural history, treatment, mutations analysis and phenotypic correlation. Liver Int. 31, 83–91 10.1111/j.1478-3231.2010.02354.x 20958917

[30] Noureen N. and Rana M.T. (2011) Neurological Wilson disease in children: a three years experience from Multan. JPMA 61, 743–748 22355993

[31] Litwin T., Dziezyc K., Karlinski M., Chabik G., Czepiel W. and Czlonkowska A. (2015) Early neurological worsening in patients with Wilson’s disease. J. Neurol. Sci. 355, 162–167 10.1016/j.jns.2015.06.010 26071888

[32] Pfeiffer R.F. (2011) Wilson’s disease. Handb. Clin. Neurol. 100, 681–709 10.1016/B978-0-444-52014-2.00049-5 21496616

[33] Bradberry S., Sheehan T. and Vale A. (2009) Use of oral dimercaptosuccinic acid (succimer) in adult patients with inorganic lead poisoning. QJM 102, 721–732 10.1093/qjmed/hcp114 19700440

[34] Guha Mazumder D.N., Ghoshal U.C., Saha J., Santra A., De B.K., Chatterjee A. et al. (1998) Randomized placebo-controlled trial of 2,3-dimercaptosuccinic acid in therapy of chronic arsenicosis due to drinking arsenic-contaminated subsoil water. J. Toxicol. 36, 683–690 9865236 10.3109/15563659809162616

[35] Lifshitz M., Hashkanazi R. and Phillip M. (1997) The effect of 2,3 dimercaptosuccinic acid in the treatment of lead poisoning in adults. Ann. Med. 29, 83–85 10.3109/07853899708998747 9073328

[36] Thurtle N., Greig J., Cooney L., Amitai Y., Ariti C., Brown M.J. et al. (2014) Description of 3,180 courses of chelation with dimercaptosuccinic acid in children </= 5 y with severe lead poisoning in Zamfara, Northern Nigeria: a retrospective analysis of programme data. PLoS Med. 11, e1001739 25291378 10.1371/journal.pmed.1001739 PMC4188566

[37] Brewer G.J., Dick R.D., Johnson V.D., Fink J.K., Kluin K.J. and Daniels S. (2001) Treatment of Wilson's disease with zinc XVI: treatment during the pediatric years. J. Lab. Clin. Med. 137, 191–198 10.1067/mlc.2001.113037 11241029

[38] Brewer G.J., Dick R.D., Yuzbasiyan-Gurkan V., Johnson V. and Wang Y. (1994) Treatment of Wilson’s disease with zinc. XIII: Therapy with zinc in presymptomatic patients from the time of diagnosis. J. Lab. Clin. Med. 123, 849–858 8201263

[39] Mizuochi T., Kimura A., Shimizu N., Nishiura H., Matsushita M. and Yoshino M. (2011) Zinc monotherapy from time of diagnosis for young pediatric patients with presymptomatic Wilson disease. J. Pediatr. Gastroenterol. Nutr. 53, 365–367 10.1097/MPG.0b013e31821d5abe 21970993

